# Seven years of telemedicine in Médecins Sans Frontières demonstrate that offering direct specialist expertise in the frontline brings clinical and educational value

**DOI:** 10.7189/jogh.08.020414

**Published:** 2018-12

**Authors:** Sophie Delaigue, Laurent Bonnardot, Olivier Steichen, Daniel Martinez Garcia, Raghu Venugopal, Jean-François Saint-Sauveur, Richard Wootton

**Affiliations:** 1 Médecins Sans Frontières, Toronto, Canada; 2 Department of Medical Ethics and Legal Medicine, Paris Descartes University, Paris, France; 3 Fondation Médecins Sans Frontières, Paris, France; 4 Department of Internal Medicine AP-HP, Hôpital Tenon, Paris, France; 5 Faculty of Medicine Sorbonne Universités, UPMC University Paris, Paris, France; 6 Médecins Sans Frontières, Geneva, Switzerland; 7 Médecins Sans Frontières, Barcelona, Spain; 8 Norwegian Centre for Integrated Care and Telemedicine, University Hospital of North Norway, Tromsø, Norway

## Abstract

**Background:**

Médecins Sans Frontières (MSF), a medical humanitarian organization, began using store-and-forward telemedicine in 2010. The aim of the present study was to describe the experience of developing a telemedicine service in low-resource settings.

**Methods:**

We studied the MSF telemedicine service during the period from 1st July 2010 until 30th June 2017. There were three consecutive phases in the development of the service, which we compared. We also examined the results of a quality assurance program which began in 2013.

**Results:**

During the study period, a total of 5646 telemedicine cases were submitted. The workload increased steadily, and the median referral rate rose from 2 to 18 cases per week. The number of hospitals submitting cases and the number of cases per hospital also increased, as did the case complexity. Despite the increased workload, the allocation time reduced from 0.9 to 0.2 hours, and the median time to answer a case decreased from 20 to 5 hours. The quality assurance scores were stable. User feedback was generally positive and more than 90% of referrers who provided a progress report about their case stated that it had been sent to an appropriate specialist, that the response was sufficiently quick and that the teleconsultation provided an educational benefit. Referrers noted a positive impact of the system on patient outcome in 39% of cases.

**Conclusions:**

The quality of the telemedicine service was maintained despite rising caseloads. The study showed that offering direct specialist expertise in low-resource settings improved the management of patients and provided additional educational value to the field physicians, thus bringing further benefits to other patients.

Médecins Sans Frontières (MSF) is an international, independent, medical humanitarian organisation that delivers emergency aid to people affected by armed conflict, epidemics, natural disasters and exclusion from health care [1]. In such contexts, medical staff must diagnose and treat patients with only limited resources, restricted referral capacity and limited ability to consult specialists.

MSF offers assistance to more than 10 million inpatients and outpatients per year [2]. In 2016, MSF provided 671 700 admissions and 9 792 200 outpatient consultations. Many patients require primary care, but some cases require specialized input.

In 2010, MSF developed a multilingual telemedicine network to assist its field medical staff by providing direct access to specialist advice (**Box 1**) [3]. The MSF telemedicine service began as a pilot project based on the Collegium Telemedicus system [4]. It uses a web-based messaging system hosted on a secure server, and store-and-forward communication. This allows a similar service to be provided to those which deliver e-consults in settings where resources are less restricted [5]. The pilot project has expanded, and at the time of the writing (August 2017) over 6000 cases have been managed by this telemedicine system.

Box 1 **The MSF Telemedicine service.**

The aim is to support MSF field workers by providing ready access to specialist opinions for multispecialty care.

There are three categories of users:

- Referrers. All MSF field health workers have access to the telemedicine service. Each MSF project site has one or more user accounts and can send cases for all situations.
- Specialists. The network of specialists is composed of MSF headquarters advisors to cover mainly paediatric, surgery, HIV/TB, infectious diseases, internal medicine, nutrition, anaesthesia and obstetrics. To reinforce this pool of specialists and for other sub-specialties not available within MSF, a network of external specialists has been built up over the years. All specialists are volunteers, from hospitals or private practice all over the world. There is a preference for those with field experience (MSF or not). All the medical and surgical specialties are represented.
- **Coordinators:** Coordinators are mainly active clinicians with MSF field experience. Two radiographers are in charge of radiological cases. They are employed to cover a definite part of the day. Each case is managed by one coordinator, who allocates the case to the most appropriate specialist and follows it until the referrer receives an appropriate answer. The coordinators decide which specialist to consult on the basis of their clinical experience. The service operates continuously, and thus coordinators are based in different world time zones.

In expanding access to the service worldwide, the challenge was to maintain or increase the quality and speed of the service. The aim of the present study was to describe the experience of developing a telemedicine service in low-resource and/or humanitarian settings, and to assess the quality and evolution of the service over seven years.

## METHODS

### Comparative study

We examined the MSF telemedicine service from 1st July 2010 until 30th June 2017. We compared three distinct periods of its development:

1. First phase: July 2010 to June 2013 (36 months). During the initiation period, case management was carried out by several different coordinators, a network of specialists was created, most of whom had no substantial telemedicine experience, and three separate networks which were operating in English, French and Spanish were merged into a single multilingual network.
2. Second phase: July 2013 to June 2015 (24 months). During the transition period, efforts were made to define procedures, develop the system (eg, a quality assurance program was introduced) and improve the governance.
3. Third phase: July 2015 to June 2017 (24 months). Telemedicine has been part of MSF's strategy since 2015. A proper administration was set up with a professional coordination pool.

We analysed various indices of network performance during these three phases, eg, allocation delay, case complexity, dialogue time (see **Box 2**). Case complexity was approximated by the numbers of messages per case, and the numbers of queries per case.

Box 2 **Principal terms used in the present paper.**

Allocation

The simplest is *one-to-one manual allocation*: a Referrer submits a new case (ie, a question about a patient), the Coordinator reads the details and decides who should reply, after which the chosen Specialist reads the details and responds to the Referrer. Many cases in a store-and-forward telemedicine system can be handled with a single consultation episode: the case is allocated to a specialist for reply, the specialist responds, and the referrer's question is answered.

Cases and queries

In practice, a single consultation episode does not always occur. For example, the Coordinator may allocate a case to a specialist who does not reply, perhaps because he or she is on holiday. In these circumstances, the Coordinator will send the case to an alternative specialist. Thus a single *case* (ie, patient) will have generated two *queries* (ie, requests to different specialists, or allocations).

Performance indicators

The allocation delay is a measure of the performance of the Coordinator(s) during the period in question. It is the interval between the arrival of the case and the first time it is allocated for reply, which is measured in hours.

The answer delay is a general measure of network performance, as perceived by the Referrer. The answer delay, which is measured in hours, is defined as the delay after a case has been submitted before the first reply is received from a Specialist. If queries are sent to several Specialists, then the answer delay is measured from case submission to the earliest reply received.

Quality assurance statistics

The quality assurance statistics measure different aspects of the network's performance. If regular quality assurance (QA) is being carried out, then five QA statistics are available:

1. process quality, *qp*
2. value to patient, *vp*
3. value to referrer, *vr*
4. value to organisation, *vo*
5. grand quality score, *gqs*.

These statistics are based on randomly-selected cases, as reviewed by the network's QA panel. Each statistic is scored on a scale from 0 = worst to 10 = best. The Grand Quality Score represents the overall network performance.

Cases were categorised as “radiological” or “clinical” based on the types of specialist consulted. The former concerned cases where the specialists were all radiologists, while clinical cases might involve a radiologist, but also involved other clinical specialists. From the telemedicine network point of view, radiological cases are simpler, because the advice is focused on image interpretation and diagnosis.

Quantitative variables were reported as median and quartiles, and temporal trends were assessed with Cuzic’s test. Categorical variables were reported as number and percentage, and temporal trends assessed with a χ^2^ for trend.

### Quality assurance

The quality assurance (QA) program began in 2013 [6-8]. Quality was measured both by evaluations made by the referrers and by a review panel:

- in the evaluation made by referrers the system automatically requests follow-up reports from the referrer. The request is sent 21 days after each case is submitted and contains 12 questions;
- in the evaluation made by a panel of qualified medical coordinators from the network, they are invited to respond independently to a questionnaire with 17 questions relating to a randomly selected case each month. This assesses (i) the quality of the allocation process and the value of the consultation for (ii) the patient, (iii) the referrer, (iv) and for MSF. Four scores are generated per panel member, from which an overall score, is calculated for the case to indicate the panel’s overall assessment. The Grand Quality Score has a range 0-10 (0=worst, 10=best). A process-control chart was used to examine the stability of the monthly Grand Quality Score over time (**Box 2**). Full details are provided in previous publications [6-8].

Ethics permission was not required, because this work was a retrospective study of non-clinical data generated during the telemedicine process, conducted by the organization’s staff in accordance with its research policies.

## RESULTS

### Comparative study

**Figure 1:**
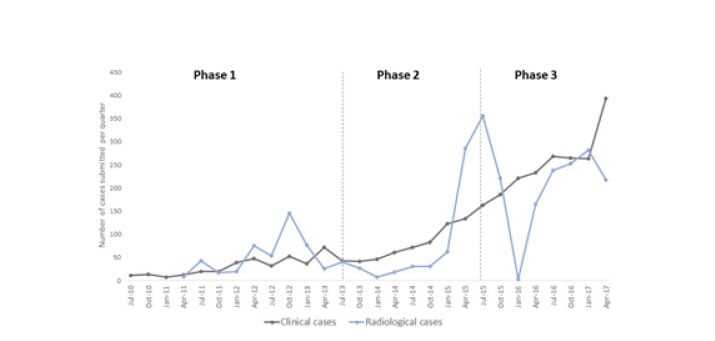
Numbers of telemedicine cases submitted per quarter, starting in July 2010. The blue line indicates Radiology-only cases; the grey line indicates General cases.

**Characteristics of cases:** From July 2010 until June 2017, a total of 5646 cases were submitted. The cases were submitted from 63 different countries. Many of those submitting large numbers of cases were countries suffering from armed conflict or internal instability (eg, South Sudan, Democratic Republic of the Congo, Yemen). The cases covered more than 54 different medical and surgical specialties. The most frequent specialties requested in terms of number of queries were paediatrics (35%), radiology (35%) and internal medicine (19%). Among the medical specialties, the most frequent subspecialties requested were infectious diseases, dermatology, neurology and tropical medicine. There was an increase in the numbers of both clinical cases and radiological cases from phase 1 to phase 3 (**Figure 1** and **Table 1**). Case complexity increased as indicated in **Table 2**.

**Table 1:** Characteristics of cases

	First phase (36 months)	Second phase (24 months)	Third phase (24 months)	*P*-value
Clinical cases, n = 2951 (52%)	360	601	1990	Not appropriate
Radiological cases, n = 2695 (48%)	465	499	1731	Not appropriate
Clinical caseload (cases/week)	2 (1-3)	5 (3-8)	18 (14-23)	<0.001
Radiological caseload (cases/week)	1 (0-4)	2 (1-5)	18 (8-24)	<0.001
Language (clinical cases)	English 265 (74%)	English 531 (88%)	English 1476 (74%)	0.01
	French 94 (26%)	French 65 (11%)	French 494 (25%)	
	Spanish 1 (0%)	Spanish 5 (1%)	Spanish 20 (1%)	
Language (radiological cases)	English 465 (100%)	English 490 (98%)	English 1719 (99%)	0.71
	French 0	French 9 (2%)	French 11 (1%)	
	Spanish 0	Spanish 0	Spanish 1 (0%)	
Proportion of paediatric cases (clinical cases)	217 (60%)	319 (53%)	1316 (66%)	<0.001
Proportion of paediatric cases (radiological cases)	151 (32%)	227 (45%)	905 (52%)	<0.001

**Table 2:** Case complexity

	First phase (36 months)	Second phase (24 months)	Third phase (24 months)	*P*-value
**Coordination complexity:**				
Coordinator messages per case clinical cases*	2 (3-5)	3 (3-5)	5 (4-8)	<0.001
Coordinator messages per case radiological cases	1 (1-1)	2 (2-2)	2 (2-2)	<0.001
**Management complexity:**				
Clinical messages per clinical case (excluding coordinator messages) clinical cases*	3 (2-5)	5 (3-7)	6 (4-9)	<0.001
Clinical messages per radiological case (excluding coordinator messages)*	2 (2-2)	3 (2-4)	2 (2-3)	0.001
Proportion of queries answered by only one specialist (clinical cases)	260 (72%)	309 (51%)	686 (34%)	<0.001
Proportion of queries answered by only one specialist (radiological cases)	438 (94%)	456 (91%)	1667 (96%)	0.003

*Median and interquartile range.

#### Field users

In 2016, MSF was active at 271 operational sites in Africa, 74 in the Middle East, 56 in Asia, 37 in Europe, 26 in the Americas and 4 in the Pacific [2]. Based on the countries of origin of cases between 2015 and 2017, the penetration of telemedicine was similar across all regions, with slightly under 50% of the hospitals in each region submitting cases.

Cases were submitted from 221 hospitals (**Table 3**). Half of the clinical cases were submitted from 24 hospitals and 90% of the radiological cases were submitted from 6 hospitals. Most of the radiological cases (70%) cases were from a single hospital based in the Central African Republic which had no radiological expertise available on-site; the lack of case submissions in January 2016 was explained by the location of the hospital in an instability region with security issues which halted its medical activities. Hospitals used the MSF telemedicine service for a median period of 67.7 weeks each (interquartile range IQR 23.3-156.6).

**Table 3:** Hospital activity

	First phase (12 quarters)	Second phase (8 quarters)	Third phase (8 quarters)	P-value
Number of hospitals submitting cases per quarter*	17 (8-23)	29 (23-33)	64 (56-70)	<0.001
Number of cases submitted by each active hospital and per quarter*	2 (1-4)	2 (1-4)	2 (1-5)	0.005

*Median and interquartile range.

#### Specialists

In parallel with the growing number of hospitals using the system, there was an increasing number of specialists; during the whole study period, 382 specialists had answered at least one case. All the specialists were either volunteers or part of the organization; 75% of specialists were from MSF headquarters or had previous MSF or low-resource field experience. Others were external experts not available within MSF, such as dermatologists, radiologists and neurologists. Most of these were based in Europe or the Americas. The specialists had a median duration of participation of 83.9 weeks each (interquartile range (IQR) = 37.5-154.5). The number of cases answered by each specialist was very heterogeneous: many had answered only a single query, while a few had answered hundreds. Half of the total queries were handled by 23 specialists: 1 dermatologist, 6 pediatricians and 16 radiologists.

#### Coordinators

An important feature of the MSF telemedicine service is the management of cases by coordinators (**Box 1**). Twelve coordinators were involved during the study period.

**Figure 2:**
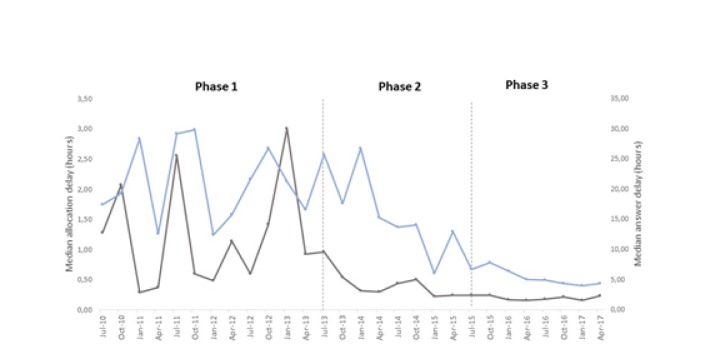
Performance indicators per quarter for general cases. The blue line indicates the answer delay and the grey line indicated the allocation delay.

**Performance indicators**: During the study, the allocation delay and answer delay were reduced even though the number of cases increased (**Figure 2**). In general, the clinical cases took longer to answer compared to radiological cases. Radiological cases were faster to allocate (**Table 4**).

**Table 4:** Performance indicators

Performance indicators*				
	**First phase (36 months)**	**Second phase (24 months)**	**Third phase (24 months)**	***P* for trend**
Allocation delay (hours): clinical cases	0.9 (0.3–3.8)	0.4 (0.1–1.0)	0.2 (0.1–0.5)	<0.001
Allocation delay (hours): radiological cases	0.4 (0.1–1.3)	0.2 (0.06–0.8)	0.1 (0.04–0.2)	<0.001
Answer delay (hours) clinical cases	20.1 (6.5–41.4)	13.3 (4.0–31.8)	5.1 (1.9–15.9)	<0.001
Answer delay (hours) radiological cases	5.8 (2.8–17.4)	7.8 (2.9–23.1)	5.4 (2.1–12.7)	<0.001

*Median and interquartile range.

### Quality assurance

#### Evaluation by referrers

During the study period, there were 526 responses to the 2325 requests for a follow-up report (23% response rate). More than 90% of respondents reported that their case had been sent to an appropriate specialist, that the response was sufficiently fast and that the teleconsultation was useful in providing an educational benefit to the referrer. However, fewer than 50% of respondents reported that an outcome improvement for the patient was likely, or that the teleconsultation was likely to improve the patient’s symptoms or function. The progress report questionnaires contained two additional free text questions, in response to which a total of 205 comments were made about the service and 229 about the case itself. Of the 205 comments about the service, the majority were positive (83%), although there were 12% negative and 5% neutral or not relevant. The majority of comments about the case were positive (49%), 13% were negative and 38% neutral or not relevant, but they were difficult to classify because referrers used this question as an additional opportunity to provide follow-up information.

#### Evaluation by review panel

During the study period, 50 randomly selected cases were reviewed by a panel of medical coordinators. The average number of panel members assessing each case was five. The median Grand Quality Score was 8 (IQR = 7.75-8.50). It remained within control limits each month, except for one case in May 2015 in which there was miscommunication between the various users involved.

## DISCUSSION

The present study summarizes the seven-year experience of the MSF telemedicine service. We found evidence that the service performed well, despite rising workloads. The first phase demonstrated the feasibility of an international program to support MSF field workers by providing ready access to specialist opinions for multispecialty care. The telemedicine system then developed to become a mature medical service characterized by improved performance indicators and stable quality assurance scores, despite increasing numbers of referring sites and increasing numbers of cases from each referring site.

Another trend we observed was the involvement of multiple specialists in the clinical cases [9]. The reasons for this include a change in the way cases were allocated, with more close case management and follow-up. At the same time, the cases tended to become more complex with more messages, more queries and more coordinator messages per case as field team members’ confidence in the network grew.

### Limitations of the study

The review panel members were all coordinators who were heavily involved in the daily operation of the service, and although their scoring was blinded, they might have been subject to unconscious bias. It would be worthwhile to develop an independent evaluation panel with diverse competences and perspectives. Another limitation was that the response rate to progress report invitations was under 30%. Without adequate feedback, the principle of requesting specialist advice is weakened because quality control is limited.

### Useful tool

The results of the present study may be compared with those from a survey of seven long-running telemedicine networks which was conducted in 2011 [10]. These networks provided humanitarian services (clinical and educational) in developing countries, and had operated for periods of 5-15 years. The number of experts serving each network ranged from 15 to 513. The smallest network had 10 requesters and the largest one had over 500 requesters. Overall, the networks operated in nearly 60 countries and had managed 1857 cases in 2011, ie, an average of 265 cases per network. Most networks had an average time to first reply of about 24 hours (range 5.6 to 72 hours). The performance of the MSF network, which has operated at a much higher workload, therefore compares favourably.

A major weakness in telehealth evaluation, at least in low and middle resource countries, is the focus on pilot studies without follow-up and rigorous evaluation. This threatens the credibility of telemedicine as a sustainable concept in these settings [11,12] The present work provides an assessment of a relatively mature service, following a previous pilot study [13] which was conducted at an earlier stage.

To our knowledge there is no comparable program of quality assurance in any other telemedicine system. Quality assurance has been conducted without interruption since 2013, as a long-term monitoring effort.

The use of increasingly specialized expertise has become the norm in high-income countries, as evidenced by the proliferation of specialties and subspecialties [14]. The expected benefits of specialized expertise are an adequate interpretation of the clinical problem and an evidence-based answer tailored to available resources [15,16]. Specialized management improves compliance with recommendations and clinical outcomes [17,18]. Although the telemedicine system aims to provide patients with the best health care possible, the benefit in terms of clinical outcome is less well documented: referrers noted a positive impact of the system on patient outcome in only 39% of cases. This may reflect the difficulties of patient follow up as much as the limited resources available to implement the specialist advice. However, the referrers stated that 93% of the consultations were useful in improving their knowledge and practice, emphasizing the educational benefit of the system. Indeed, many local doctors had limited access to practical education in medical and surgical specialties during their training and the system mitigates this deficiency. By providing answers to difficult clinical questions, the system reduces field physicians resignation and practice impoverishment. This observation is in line with recent evidence showing the importance of experiential learning to build clinical expertise and judgment [19].

### Barriers to adoption and elements of success

The results of implementing a program of this type are not immediate, achieving only modest benefits in the early years. As has been observed elsewhere, the introduction of telemedicine comes with multiple obstacles and challenges [20], and after seven years the MSF telemedicine service has encountered many of them:

-Technical: Internet connectivity is challenging in many remote areas where MSF operates, and is often affected by service interruptions such as power outages or irregular connection.
-Operational: the high turnover of field users makes it difficult to train new potential users and to monitor the use of the service.
-Cultural: use of telemedicine represents a change in the clinical process and requires the development of trust between health professionals in numerous countries.

The choice of a store-and-forward system was partly made to address these issues. Indeed, real time communication is not only expensive [21] but more vulnerable to technical problems [22] and less flexible for physicians with busy schedules. As a result, store-and-forward systems appear to be more appropriate than real time systems in resource-limited settings [23,24], although both approaches can be combined.

Factors which have supported the consolidation and the sustainability of the service include:

- Ongoing maintenance and development of the portal based on the QA program to improve the service with a constant flow between innovation and evaluation [25]. A total of 14 peer-reviewed publications have been produced by the members of the organization, providing strong evidence of the effectiveness, performance, quality and sustainability of the service.
- A stable medical coordination team during the third phase of the study has improved the follow-up of cases and reinforced the trust of users by ensuring optimal exchanges between referrers and specialists [26].
- The spread of mobile phones [27] has probably enhanced the efficiency of coordinator and specialist interactions, and the availability of a mobile application would benefit the field health workers alike.

### Next steps

The usual life-cycle of a telemedicine project can be characterised by four stages: technical development, implementation, integration at a larger scale, and then maintenance with the use of the service in routine patient care [28]. The first part of this life-cycle corresponds to the three phases of MSF telemedicine that we have observed. However, integration at a larger scale has not yet occurred in MSF. For example, in 2016 more than 10 million patients were treated by MSF, while only 1644 telemedicine cases (0.02%) were submitted for a specialist opinion. Although there are no data for low-resource settings, we know that 5%-8% of general practice consultations require specialist consultation in high income countries [29,30]. It therefore seems likely that more MSF patients could benefit from specialist telemedicine expertise.

Not only is further adoption of telemedicine itself required across MSF, the consolidation of the QA activities into a comprehensive QA is necessary in order to achieve institutionalization of the work, and to realise its full potential [31]. Although this might incur costs in the short-term, the increased efficiency and effectiveness could lead to net savings for the organisation in the long-term.

## CONCLUSIONS

The telemedicine service developed by Médecins Sans Frontières has gradually been adopted within the organisation over a seven-year period. The quality of the service has been maintained despite rising caseloads. By offering direct specialist access in low-resource settings patient management is improved and there is additional long-term educational value for field physicians.
